# Expression of circ-PHC3 enhances ovarian cancer progression via regulation of the miR-497-5p/SOX9 pathway

**DOI:** 10.1186/s13048-023-01170-w

**Published:** 2023-07-20

**Authors:** Hongxia Wang, Suwei Lan, Lingxiang Wang, Jingyun Zhao, Xinzhuan Jia, Jie Xu, Guangyu Sun, Leilei Liu, Shan Gong, Na Wang, Baoen Shan, Fenghua Zhang, Zhengmao Zhang

**Affiliations:** 1 Department of Gynecology, Fourth Hospital of Hebei Medical University, No.12 Jiankang Road, Shijiazhuang, 050011 China; 2 Department of Reproductive Medicine, Fourth Hospital of Hebei Medical University, Shijiazhuang, China; 3 Department of Gynecology, Third Hospital of Hebei Medical University, Shijiazhuang, China; 4 Research Center, Fourth Hospital of Hebei Medical University, Shijiazhuang, China; 5 Department of Breast & Thyroid Surgery, Hebei General Hospital, No.348 Heping West Road, Shijiazhuang, 050051 Hebei China

**Keywords:** Circ-PHC3, Ovarian cancer, SOX9, miR-497-5p, Cancer stem cell

## Abstract

**Background:**

Accumulating studies have reported indispensable functions of circular RNAs (circRNA) in tumor progression through regulation of gene expression. However, circRNA expression profiles and functions in human ovarian carcinoma (OC) are yet to be fully established.

**Methods:**

In this research, deep sequencing of circRNAs from OC samples and paired adjacent normal tissues was performed to establish expression profiles and circ-PHC3 levels between the groups further compared using RT-qPCR. The effects of ectopic overexpression of miR-497-5p and SOX9 and siRNA-mediated knockdown of circ-PHC3 and an miR-497-5p inhibitor were explored to clarify the regulatory mechanisms underlying circ-PHC3 activity in OC proliferation and metastasis. Information from public databases and the luciferase reporter assay were further utilized to examine the potential correlations among circ-PHC3, miR-497-5p and SOX9.

**Results:**

Our results showed significant upregulation of circ-PHC3 in both OC cell lines and tissues. In the luciferase reporter assay, downregulation of circ-PHC3 led to suppression of metastasis and proliferation, potentially through targeted effects on the miR-497-5p/SOX9 axis in OC. SOX9 overexpression or miR-497-5p suppression rescued OC cell proliferation and invasion following silencing of circ-PHC3. Moreover, SOX9 inhibition induced restoration of OC cell invasion and proliferation under conditions of overexpression of miR-497-5p. Thus, circ-PHC3 appears to exert effects on cancer stem cell differentiation through regulation of the miR-497-5p/SOX9 axis.

**Conclusion:**

Taken together, our findings suggest that circ-PHC3 enhances OC progression through functioning as an miR-497-5p sponge to promote SOX9 expression, supporting its potential as a promising candidate target for OC therapy.

**Supplementary Information:**

The online version contains supplementary material available at 10.1186/s13048-023-01170-w.

## Introduction

Ovarian cancer (OC) is among the most common gynecological malignancies and a significant contributory factor to cancer-related mortality in women [1]. Survival rates remain low, with only ~ 20% advanced-stage OC cases surviving beyond 12 years post-treatment [2]. OC is classified into ovarian surface epithelium and serous intra-epithelial carcinoma categories. The main treatment option is debulking surgery along with platinum-based chemotherapy. The success rate of debulking surgery is based on the surgical techniques and identification of tumor gene signatures [3], highlighting the importance of effective biomarkers for early diagnosis.

Several investigations to date have revealed key functions of non-coding RNAs (ncRNAs) in tumorigenesis [4, 5]. Circular RNAs (circRNAs) are a type of ncRNA constituting a series of conserved endogenous RNAs generated by exon skipping or back-splicing events, which regulate gene expression via competitive binding to microRNAs (miRNA) [6]. For instance, hsa_circ_0078607 suppresses OC progression through regulation of the miR-518a-5p/Fas signaling pathway [7] while circ-PGAM1 enhances malignant development of epithelial OC through effects on the miR-542-3p/CDC5L/PEAK1 pathway [7]. However, the circRNA expression profiles and functions in human ovarian carcinoma are yet to be established.

In the current study, circRNA transcripts were characterized based on RNA sequencing (RNA-seq) of ribosomal RNA-depleted total RNA from OC and adjacent non-cancerous tissue samples. Our results showed high expression of hsa_circ_0005228 (circ-PHC3) in OC tissues, distinct from other tumor types. Hsa_circ_0005228 originates from back-splicing of the exonic PHC3 gene. The sample size was subsequently expanded for comprehensive analysis of circ-PHC3 expression, which confirmed a significant increase in OC relative to adjacent non-tumorous tissues. To explain this finding, the functions and underlying mechanisms of circ-PHC3 in OC were further explored. Based on the collective results, we propose that circ-PHC3 functions as an miR-497-5p sponge to enhance SOX9 expression, in turn, promoting invasion and proliferation of OC cells.

## Materials and methods

### Animal ethics statement

Four-week-old BALB/c female nude mice weighing 15–20 g (Shanghai Jihui Laboratory Animal Care Co., Ltd.) were used in this study. All animal experiments were approved by The Ethics Committee of the Fourth Hospital of Hebei Medical University. Experiments were conducted according to the Guide for Care and Use of Laboratory Animals (8^th^ Edition).

### Tissue samples

We collected 10 fresh OC and adjacent noncancerous OC tissues with informed consent from patients in the Fourth Hospital of Hebei Medical University. Patients did not receive chemotherapy or radiotherapy prior to tissue sampling. Samples were snap-frozen in liquid nitrogen and maintained at -80 °C until RNA extraction. The Ethics committee from the Fourth Hospital of Hebei Medical University approved our research.

### Strand-specific RNA-seq library preparation and high-throughput RNA-Seq

Total RNA was extracted from OC and paired adjacent noncancerous OC tissues using TRIzol reagent (Invitrogen, Carlsbad, CA, USA). Tissues were treated with DNase I (DNA-free kit, Ambion, Texas, USA) twice at 37 °C for 0.5 h each time. Total RNA (~ 3 μg) from each sample was treated with the VAHTS total RNA-seq (H/M/R) Library Prep Kit for Illumina (Vazyme Biotech Co., Ltd, Nanjing, China) to remove ribosomal RNA while retaining other RNA types (including mRNAs and ncRNAs). RNAs were purified using RNase R (Epicenter; 40 U) at 37 °C for 3 h, followed by treatment with TRIzol. Subsequently, an RNA-seq library using the KAPA Stranded RNA-Seq Library Prep Kit (Illumina) was constructed and subjected to deep sequencing with Illumina HiSeq 4000 (Aksomics, Inc., Shanghai, China).

#### Cell culture

We purchased the human ovarian epithelial cell line, IOSE80, from Shanghai Zhen Biotechnology Co., Ltd. (Shanghai, China) and two OC cell lines, SKOV3 and OV90, from American Type Culture Collection (ATCC, Manassas, VA, USA). All cells were cultured in RPMI-1640 (cat no. SH30809.01; Hyclone, Logan, UT, USA) with 10% fetal bovine serum (FBS, cat no. SH30084.03; Hyclone) and 5% CO_2_ at 37 °C.

#### Cell transfection

Circ-PHC3 small interfering RNA (si-circ-PHC3, 5’-CUCAGAUUUAAGGACCAUAUU-3’), miR-497-5p inhibitor (5’-ACAAACCACAGUGUGCUGCUG-3’), miR-497-5p mimic (5’-CAGCAGCACACUGUGGUUUGUAAACCACAGUGUGCUGCUGUU-3’), SOX9 overexpression vector (SOX9) and the respective negative controls (NC) were synthesized by GenePharma (Shanghai, China). Cell transfection experiments were performed at ~ 80% confluence using Lipofectamine 3000 (cat no. L3000-015; Invitrogen).

#### Bioinformatics analysis

Bioinformatics analysis was conducted to predict circRNA/miRNA target genes through https://circinteractome.nia.nih.gov/. Correlations of miR-497-5p and SOX9 were predicted using http://www.targetscan.org/ and circ-PHC3 expression and OC patient prognosis predicted using http://gepia.cancer-pku.cn/.

### Quantitative real-time polymerase chain reaction (qPCR)

Total RNA was extracted from cancer tissue and cells using TRIzol reagent (Invitrogen). cDNA was synthesized and amplified using the TaqMan miRNA Reverse Transcription Kit (Thermo Fisher Scientific) and qPCR conducted with the TaqMan™ MicroRNA Assay Kit (Applied Biosystems, Foster City, CA, USA). We applied the 2^−ΔΔCT^ method to detect related fold changes in expression, with *GAPDH* and *U6* as the internal references. The following primers were utilized: circ-PHC3 forward, 5'-GGCTGCTGTACAGTC-3', reverse, 5'-GTGAGGTGGTGGTG-3'; miR-497-5p forward, 5'-CCTTCAGCAGCACACTGTGG-3', reverse 5'-CAGTGCAGGGTCCGAGGTAT-3'; *SOX9* forward, 5'-TGAAGATGACCGACGAGCAGGAGAAG-3', reverse, 5'-CTTCCTCGCTCTCCTTCTTCAG-3'; U6 forward, 5'-CTCGCTTCGGCAGCACA-3', reverse 5'-AACGCTTCACGAATTTGCGT-3'; *GAPDH* forward 5'-AATGGGCAGCCGTTAGGAAA-3', reverse 5'-TGAAGGGGTCATTGATGGCA-3'.

#### Plate colony formation assay

SKOV3 and OV90 cells subjected to various treatments were used to generate cell suspensions. To this end, we transferred 200 cells to 6-well plates, which were cultured in an incubator for 10 d at 37 °C prior to morphological analysis. The culture medium was changed every 3 days. Prior to the end of the experiment, our team observed images of cells under a light microscope (Primovert; Carl Zeiss, Jena, Germany), which were subsequently washed twice using phosphate-buffered saline (PBS). Next, 500 µL Giemsa dye was added to individual wells. Cells were stained for 10–20 min followed by three washes with double-distilled water. Images were captured using a digital camera.

#### CCK-8 assay

Cells were incubated in 10% CCK-8 solution diluted with normal culture medium at 37 °C, which resulted in a color change. Cell proliferation rates at 1, 2, and 3 d were assessed following transfection via measurement of absorbance at 570 nm using a microplate reader.

#### Transwell migration assay

Transwell chambers (Corning, NY, USA) were utilized for transwell migration and matrigel invasion assays conducted following standard protocols (BD Biosciences, Bedford, MA, USA). In brief, 200 μL serum-free medium including 5 × 10^4^ treated cells was transferred to the upper chambers and 600 μL complete medium added to the lower chambers. After incubation of cells for 1 day, images were obtained under an inverted light microscope (Primovert; Carl Zeiss, Jena, Germany). Migrated cells were quantified from more than two random fields of view.

#### Tumor sphere formation assay

Single OV90 and SKOV3 cells were resuspended in serum-free DMEM medium. Following cell sorting, 200 cells/well in 200 μL serum-free medium supplemented with 20 ng/mL bFGF and 20 ng/mL EGF were cultured in 96-well plates at a density of 10 wells/group, with medium changes every two days. Five regions were randomly selected per well with the aid of a camera-equipped microplate reader (Leica, Wetzlar, Germany). The sphere percentage was expressed as sphere number/200.

#### Dual-luciferase reporter assay

The putative miR-497-5p binding site was cloned into *SOX9* 3'-UTR and wild-type (WT) or mutant (MUT) circ-PHC3 into psi-CHECK vector (Promega, Madison, WI USA) downstream of firefly luciferase 3'-UTR or circ-PHC3 as the primary luciferase signal using Renilla luciferase as the normalization signal, and the constructs designated SOX9-Wt/circ-PHC3-Wt and SOX9-Mut/circ-PHC3-Mut, respectively. The psi-CHECK vector normalized the Renilla luciferase signal to compensate for variations between harvested efficiencies and transfections. Lipofectamine 2000 (Invitrogen Life Technologies) was employed for transfection into HEK293 cells. We examined Renilla and firefly luciferase activities one day after transfection using the Dual-Luciferase Reporter Assay System (Promega, Mannheim, Germany) and a luminometer (Molecular Devices, San Jose, CA, USA). Relative Renilla luciferase activity was calculated following the manufacturer's instructions.

### In vivo experiments

Animal experiments were performed in accordance with established procedures (8). To validate the nude mouse model of OC, stable lentiviral-mediated circ-PHC3-silenced (sh-circ-PHC3) (1 × 10^6^) SKOV3 cells or NC in 100 μL PBS were injected into flanks of BALB/C nude mice, and tumor volumes and weights were measured. Each treatment group contained six mice.

For analysis of tumor metastasis, stable lentiviral-mediated circ-PHC3-silenced (sh-circ-PHC3) (2 × 10^5^) SKOV3 cells or NC in 100 μL PBS were injected into tail veins of 4 week-old female nude mice. After 4 weeks, lung metastasis was evaluated using an in vivo bioluminescence imaging system. The number of metastatic foci in lung tissues was assessed via hematoxylin and eosin staining.

### Immunofluorescence and immunohistochemistry

Tumor tissue samples were fixed in 10% formalin solution, followed by embedding in paraffin. Sections (5 μm) were subsequently stained with Ki-67 for evaluation of proliferation. Samples were examined under an Axiophot light microscope (Zeiss, Oberkochen, Germany) and images captured using a digital camera.

### Statistical analysis

Data are shown as means ± standard deviation (SD). Statistical analyses were performed in GraphPad Prism (La Jolla, USA) to assess significant differences among groups. *P*-values ≤ 0.05 were regarded as statistically significant. Two-tailed Student’s *t*-tests were used to determine significant differences between two groups, and one-way ANOVA with post hoc Bonferroni test was used to determine significant differences among three or more groups.

## Results

### Circ-PHC3 is upregulated in OC cells and tissues

**Fig. 1:**
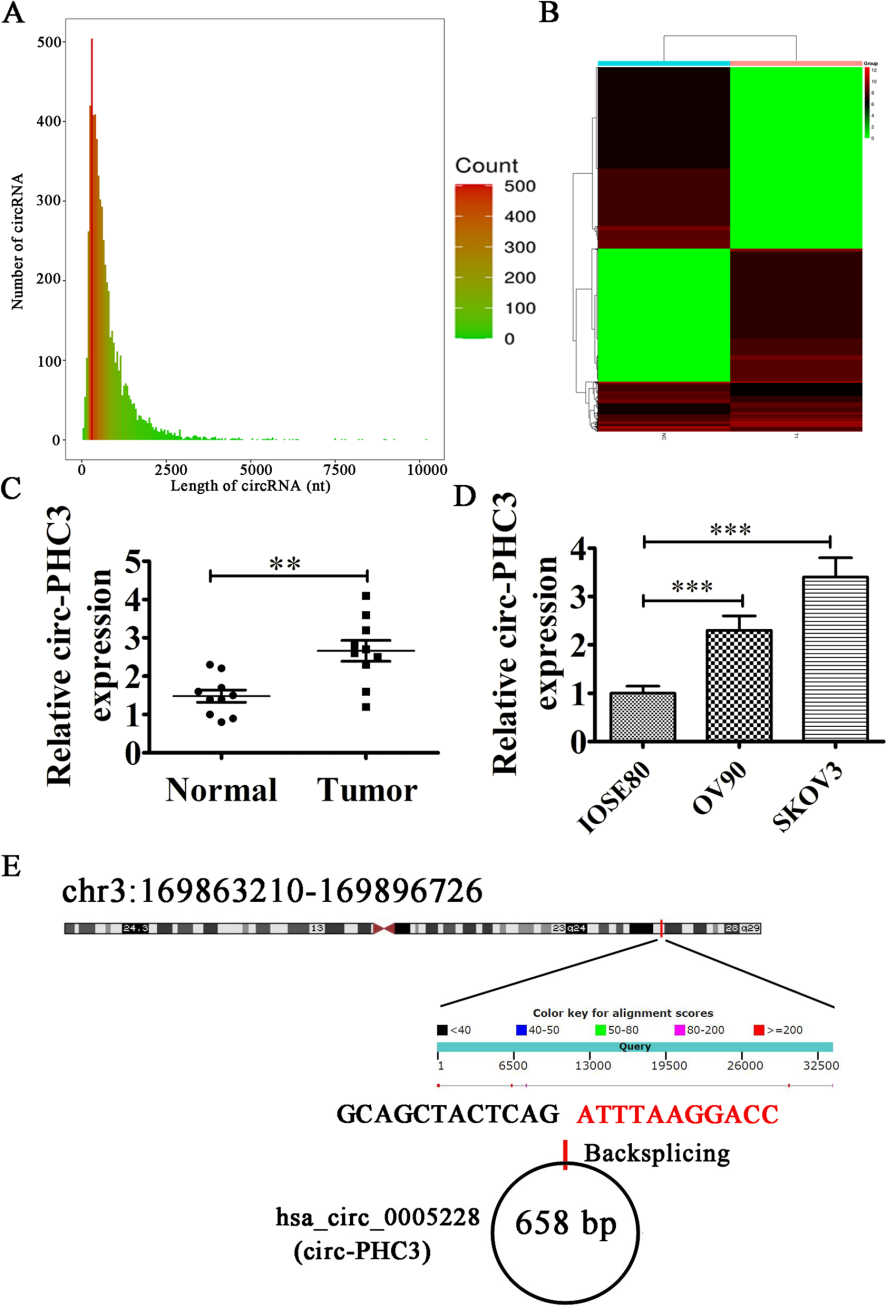
Circ-PHC3 is upregulated in OC tissues and cells. **A** Length distribution of the identified circRNAs (X-axis: lengths of circRNAs identified in this study, Y-axis: abundance of circRNAs classified by different lengths). **B** Heat map of all differentially expressed circRNAs between normal and tumor tissues. **C** qRT-PCR analysis of circ-PHC3 expression in OC tissues (*n* = 10) and adjacent normal tissues (*n* = 10). Data are presented as means ± SD. **P < 0.01. **D** RT-qPCR analysis of relative circ-PHC3 levels in OC (OV90 and SKOV3) and normal ovarian epithelial (IOSE80) cells. Data are presented as means ± SD. ***P < 0.001. **E** Genomic loci of circ-PHC3 and PHC3

Our results support an association of abnormal circRNA expression with progression of OC. RNA-seq analysis of ribosomal RNA-depleted total RNA from OC tissue and adjacent normal samples was conducted and scatter plots generated to compare circRNA expression patterns between matching OC and non-tumor tissues. The majority of circRNAs identified were < 1500 nucleotides (nt) (Fig. 1A). We observed significant differences in expression patterns of specific circRNAs, such as circ-PHC3, between tumor and non-tumor tissue counterparts (Fig. 1B, Supplementary materials). To clarify the role of circ-PHC3, expression of this circRNA in OC was further examined via qRT-PCR. The experiments confirmed upregulation of circ-PHC3 in OC tissues compared with matched non-tumor tissues, consistent with RNA-seq data (Fig. 1C). The significant increase in circ-PHC3 expression in OC cell lines (OV90 and SKOV3) relative to the normal ovarian epithelial cell line IOSE80 (Fig. 1D) suggests an oncogenic role of this circRNA. Circ-PHC3 (hsa_circ_0005228) originates from cyclization of 5' exons from the PHC3 gene at chr3:169,863,210–169,896,726. The PHC3 transcript is 33,516 bp while the spliced mature circRNA is 658 bp in length (Fig. 1E). Accordingly, we designated hsa_circ_0005228 as circ-PHC3.

### Circ-PHC3 knockdown suppresses OC cell proliferation and growth in vivo and in vitro

**Fig. 2:**
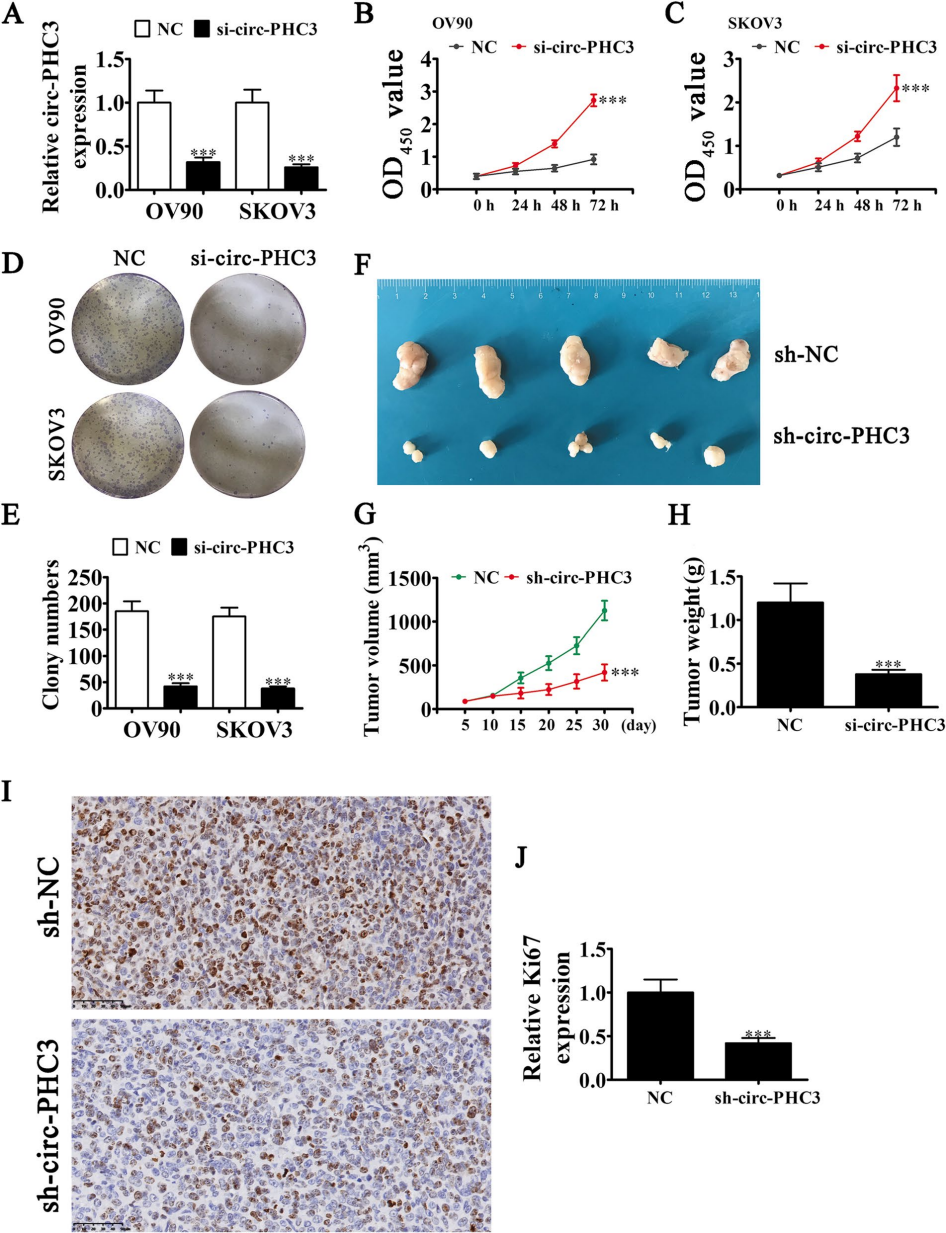
Knockdown of circ-PHC3 inhibits proliferation and growth of OC cells in vivo and in vitro. **A** qRT-PCR detection of circ-PHC3 expression in OV90 and SKOV3 cells after transfection with negative control (NC) or specific siRNA against circ-PHC3. Data are presented as means ± SD. ***P < 0.001 vs NC. **B**, **C** Assessment of cell proliferation via the CCK-8 assay. Data are presented as means ± SD. ***P < 0.001 vs NC. **D**, **E** Assessment of OV90 and SKOV3 cell proliferation using the clone formation assay. Data are presented as means ± SD. ***P < 0.001 vs NC. **F** Representative images of nude mouse xenografts after injection of SKOV3 cells transfected with sh-NC or sh-circ-PHC3. **G** Data on tumor growth measured every 5 days are presented as means ± SD. ***P < 0.001 vs sh-NC. **H** Data on tumor weight measured 30 days after grafting are presented as means ± SD. ***P < 0.001 vs sh-NC. **I**, **J** Immunohistochemical staining showing the expression of Ki-67 in tumor specimens from the sh-NC and sh-circ-PHC3 groups. Data are expressed as means ± SD. ***P < 0.001 vs sh-NC

To reveal the effects of circ-PHC3 on the proliferation and tumor growth of OC, we generated siRNA against circ-PHC3 for transfection into OV90 and SKOV3 cells. RT-qPCR analysis validated a significant decrease in circ-PHC3 expression in SKOV3 and OV90 cells compared with NC and control groups (Fig. 2A). Data from CCK-8 (Fig. 2B-C) and colony formation assays (Fig. 2D-E) confirmed that circ-PHC3 silencing inhibited proliferation of OV90 and SKOV3 cells. In an in vivo xenograft mouse model, tumor volumes (Fig. 2F, G) and weights (Fig. 2H) of nude mice injected with SKOV3 cells transfected with circ-PHC3 siRNA were decreased. Immunohistochemical Ki67 staining showed inhibition of Ki67 expression under conditions of circ-PHC3 silencing (Fig. 2I, J), clearly indicating that downregulation of circ-PHC3 suppresses OC cell proliferation and tumor growth.

### Downregulation of circ-PHC3 inhibits metastasis

**Fig. 3:**
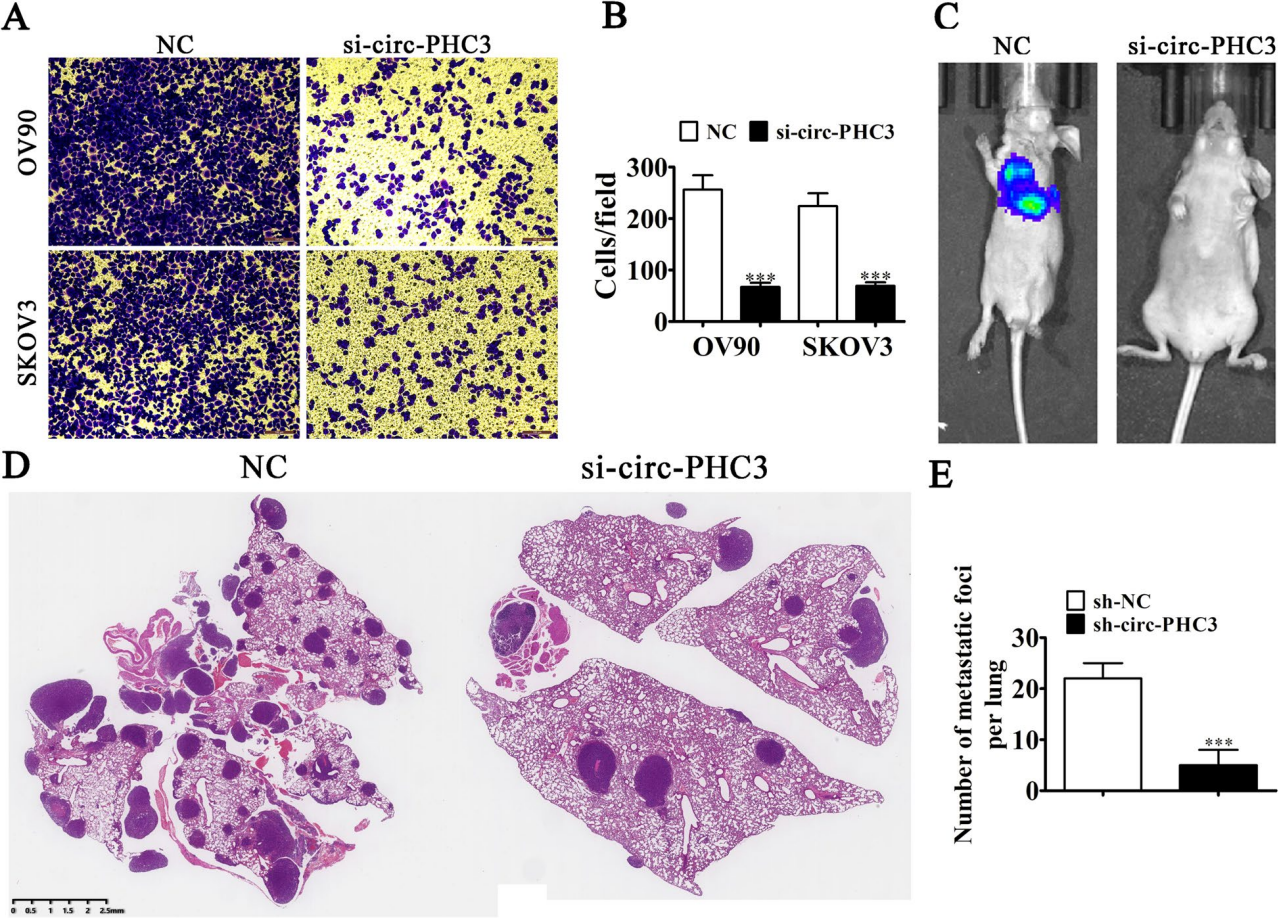
Downregulation of circ-PHC3 inhibits metastasis. **A**, **B** Transwell detection of the migration abilities of OV90 and SKOV3 cells with and without circ-PHC3 silencing. Data are presented as means ± SD. ***P < 0.001 vs NC. **C** Live imaging showing the effects of circ-PHC3 on metastasis of SKOV3 OC cells after intravenous tail injection for 4 weeks. **D**, **E** Number of metastatic foci in lung tissues calculated based on HE staining. Data are expressed as mean ± SD. ^***^P < 0.001 vs sh-NC

To reveal the effects of circ-PHC3 on the metastasis of OC, we constructed a circ-PHC3 silenced vector, which was transfected into both OV90 and SKOV3 cells. In the transwell assay, downregulation of circ-PHC3 suppressed migration of both OV90 and SKOV3 cell lines (Fig. 3A, B). Live imaging revealed involvement of circPHC3 in pulmonary metastasis of SKOV3 cells. Consistently, circ-PHC3 silencing induced a decrease in pulmonary metastasis, as indicated by reduced metastatic foci numbers in lung tissues visualized via hematoxylin and eosin staining (Fig. 3C-E). Our findings confirmed that downregulation of circ-PHC3 induces suppression of invasive OC cell activity.

### miR-497-5p and SOX9 are downstream targets of circ-PHC3

**Fig. 4:**
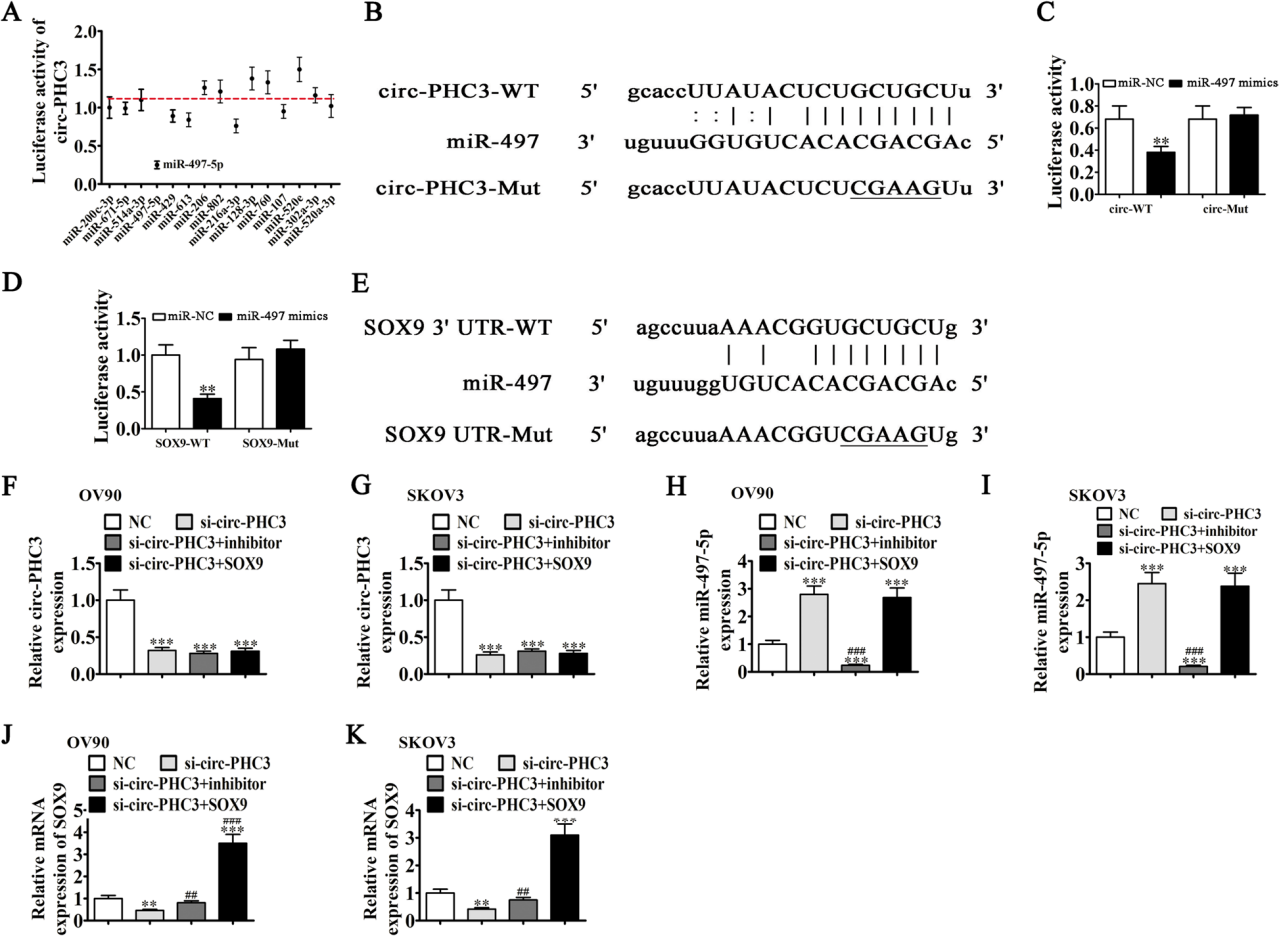
miR-497-5p and SOX9 are downstream targets of circ-PHC3. **A** Luciferase activity was normalized to that of Renilla luciferase. **B** Predicted binding sites of miR-497-5p in circ-PHC3. Both WT and MUT versions of circ-PHC3 are presented. **C** Relative luciferase activity determined 48 h after transfection of HEK293T cells with miR-497-5p mimic/NC or circ-PHC3 WT/Mut. Data are presented as means ± SD. **P < 0.01. **D** Predicted binding sites of miR-497-5p within the 3'UTR of SOX9. Both WT and MUT versions of 3'-UTR-SOX9 are shown. **E** Relative luciferase activity determined 48 h after transfection of HEK293T cells with miR-497-5p mimic/NC or 3'UTR-SOX93 WT/Mut. Data are presented as means ± SD. **P < 0.01. (F-K) RT-qPCR detection of expression of circ-PHC3, miR-497-5p and SOX9 after transfection with circ-PHC3 (**F**, **G**), miR-497-5p (**H**, **I**) and SOX9 (**J**, **K**) siRNA or overexpression vectors, either singly or in combination, in OV90 and SKOV3 cells. Data are presented as means ± SD; ^**^P < 0.01, ^***^P < 0.001 vs NC; ^##^*P* < 0.01, ^###^*P* < 0.001 vs si-circ-PHC3

To reveal the downstream target of circ-PHC3, we performed bioinformatics analyses, which indicated interactions of circ-PHC3 with several miRNAs, including miR-200c-3p, miR-671-5p, miR-514a-3p, miR-497-5p, miR-613, miR-206, and miR-760. In HEK293 cells transfected with luciferase reporter vector containing the circ-PHC3 sequence and various miRNA mimics, miR-497-5p induced a significant decrease in fluorescence intensity, supporting the conclusion that this miRNA is a downstream target of circ-PHC3 (Fig. 4A). Luciferase reporter experiments illustrated suppression of luciferase activity in miR-497-5p WT but not MUT cell lines (Fig. 4B, C), further confirming the validity of miR-497-5p as a circ-PHC3 target.

Earlier bioinformatics analyses identified SOX9 as a downstream target of miR-497-5p. To explore the potential correlation between SOX9 and miR-497-5p, 3'-UTR-SOX9 constructs with WT or MUT miR-497–5 binding sequences were inserted into the luciferase reporter vector, which was transfected into HEK293 cells with or without miR-497-5p mimic. Luciferase reporter analyses showed that miR-497-5p suppressed luciferase activity in WT but not MUT-transfected cell lines (Fig. 4D, E), clearly inferring that SOX9 is a miR-497-5p target.

RT-qPCR data showed a decrease in circ-PHC3 expression after transfection of cells with circ-PHC3-silenced vector. Notably, miR-497-5p inhibitor treatment and SOX9 overexpression had no influence on circ-PHC3 expression in both OV90 and SKOV3 cell lines (Fig. 4F, G), suggesting that miR-497-5p and SOX9 act downstream of circ-PHC3, as expected. Circ-PHC3 silencing led to upregulation of miR-497-5p. Overexpression of SOX9 did not affect circ-PHC3-induced regulation of miR-497-5p expression (Fig. 4H, I), supporting the theory that miR-497-5p is a downstream target of circ-PHC3. Moreover, knockdown of circ-PHC3 suppressed SOX9 expression while inhibition of miR-497-5p restored SOX9 expression under circ-PHC3 silencing. Following transfection with SOX9 overexpression vector, SOX9 expression was significantly increased (Fig. 4J, K). Our collective data suggest that circ-PHC3 enhances SOX9 expression via miR-497-5p sponging.

### SOX9 overexpression or miR-497-5p inhibition rescues OC cell proliferation and migration patterns under silencing of circ-PHC3

**Fig. 5:**
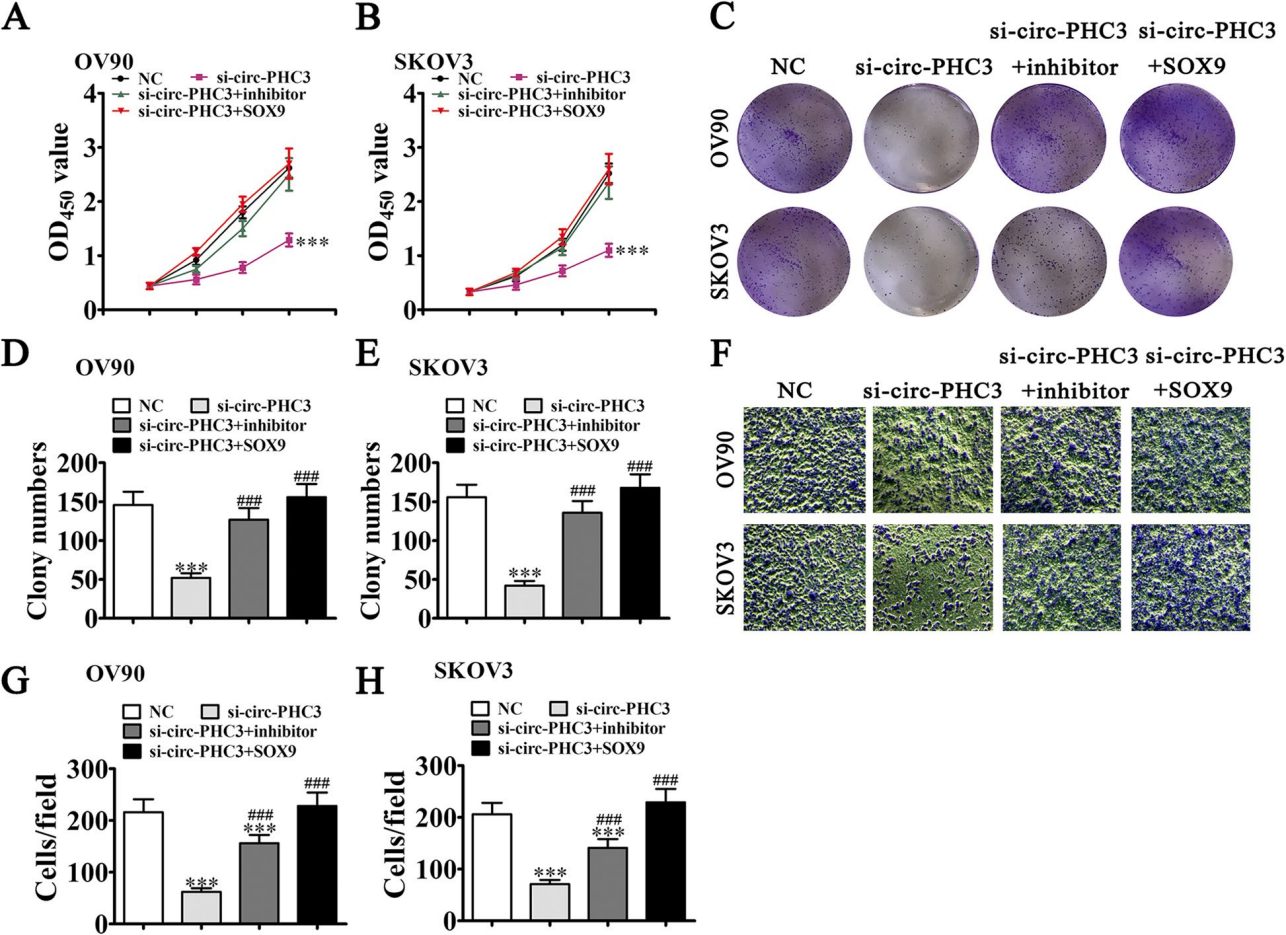
Overexpression of SOX9 or inhibition of miR-497-5p rescues OC cell proliferation and migration under silencing of circ-PHC3. **A**, **B** CCK-8 assay showing the proliferative ability of OV90 and SKOV3 cells. Data are expressed as means ± SD. ^***^P < 0.001 vs NC. **C**-**E** Clone formation assays showing the proliferation ability of OV90 and SKOV3 cells. Data are expressed as means ± SD. ^***^P < 0.001 vs NC. ^###^*P* < 0.001 vs. si-circ-PHC3. **F**–**H** Transwell detection of the invasion and migration abilities of OV90 and SKOV3 cells. Data are expressed as means ± SD. ^***^P < 0.001 vs NC. ^###^*P* < 0.001 vs. si-circ-PHC3

To reveal the regulatory relationships among circ-PHC3, miR-497-5p, and SOX9, we constructed circ-PHC3, miR-497-5p, and SOX9 overexpression and silencing vectors, and transfected them into both SKOV3 and OV90 cells. CCK-8 (Fig. 5A, B) and clone formation (Fig. 5C-E) assays demonstrated that miR-497-5p suppression and SOX9 overexpression rescued proliferation of both OV90 and SKOV3 cell lines under conditions of circ-PHC3 silencing. Similarly, in the transwell assay, SOX9 overexpression and miR-497-5p suppression restored migration of both OV90 and SKOV3 cells under conditions of circ-PHC3 knockdown (Fig. 5F-H).

### Overexpression of SOX9 rescues OC cell proliferation and migration patterns under miR-497-5p overexpression

**Fig. 6:**
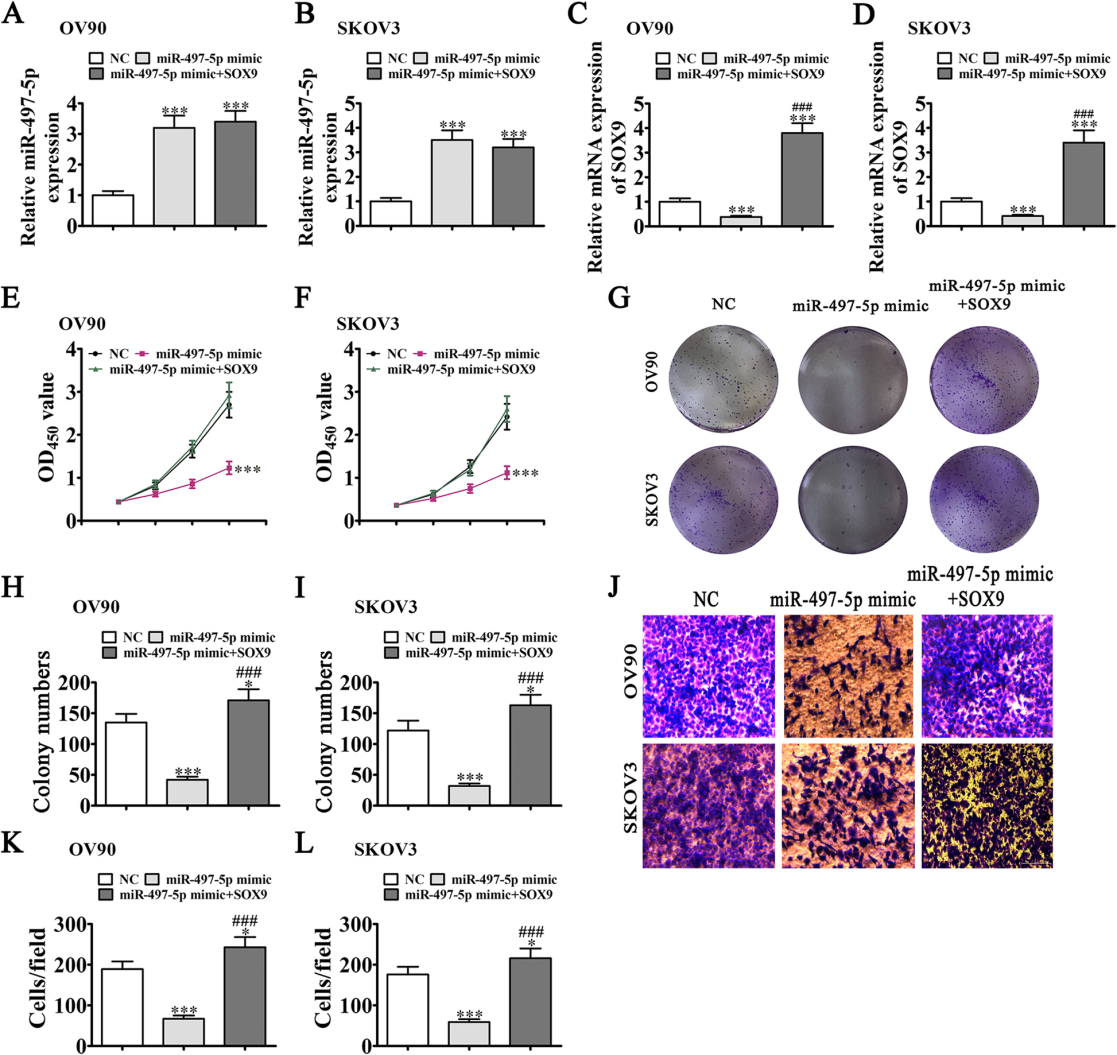
Overexpression of SOX9 rescues OC cell proliferation and migration under conditions of miR-497-5p overexpression. **A**-**D** RT-qPCR showing expression patterns of miR-497-5p and SOX9 in OV90 and SKOV3 cells. **E**, **F** CCK-8 assay of the proliferation ability of OV90 and SKOV3 cells. Data are expressed as means ± SD. ^***^P < 0.001 vs NC. **G**-**I** Clone formation assay showing the proliferation ability of OV90 and SKOV3 cells. Data are expressed as means ± SD. ^*^P < 0.05, ^***^P < 0.001 vs NC. ^###^*P* < 0.001 vs. miR-497-5p mimic. **J**-**L** Transwell detection assay of the invasion and migration capabilities of OV90 and SKOV3 cells. Data are expressed as means ± SD. ^*^P < 0.05, ^***^P < 0.001 vs NC. ^###^*P* < 0.001 vs miR-497-5p mimic

To reveal the regulatory relationship between miR-497-5p and SOX9, we constructed miR-497-5p and SOX9 overexpression vectors, and transfected them into both SKOV3 and OV90 cells. RT-qPCR experiments showed miR-497-5p overexpression in SKOV3 and OV90 cells following transfection with the miR-497-5p mimic (Fig. 6A, B). Notably, miR-497-5p overexpression induced inhibition of SOX9 expression. Under conditions of SOX9 overexpression, SOX9 levels were restored, even after miR-497-5p upregulation (Fig. 6C, D), indicating that SOX9 acts downstream of miR-497-5p. CCK-8 (Fig. 6E, F) and clone formation (Fig. 6G-I) assays demonstrated that SOX9 overexpression rescued OV90 and SKOV3 cell proliferation following miR-497-5p upregulation, consistent with migration data from the transwell assay (Fig. 6J-L).

### Circ-PHC3 influences cancer stem cells differentially via regulation of miR-497-5p/SOX9

**Fig. 7:**
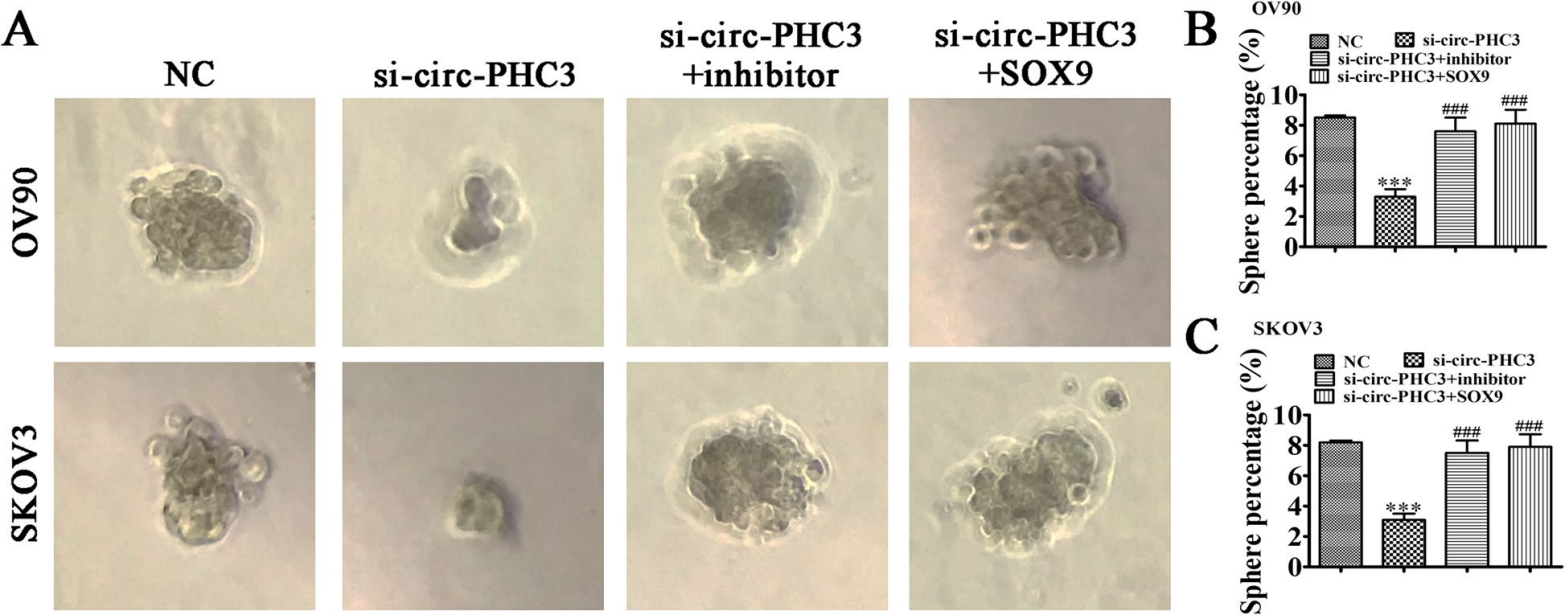
Circ-PHC3 influences cancer stem cells differentially via regulation of the miR-497-5p/SOX9 axis. **A**-**C** Images of tumor sphere formation in OV90 and SKOV3 cells. Data are expressed as means ± SD. ^*^P < 0.05, ^***^P < 0.001 vs NC. ^###^*P* < 0.001 vs miR-497-5p mimic

To reveal the effects of circ-PHC3 on tumor stem cell differentiation, we performed tumor sphere formation assays in both SKOV3 and OV90 cells. The results indicated that circ-PHC3 silencing induced a decrease in cell division. Notably, SOX9 overexpression or miR-497-5p inhibition led to recovery of tumor sphere formation ability (Fig. 7). The collective findings support the theory that circ-PHC3 influences cancer stem cell differentiation through regulation of the miR-497-5p/SOX9 axis.

## Discussion

OC is a common gynecologic malignancy often presenting at advanced stages with unsatisfactory prognosis. Improved understanding of the pathways underlying tumor progression should aid in the development of efficient OC treatment strategies.

The physiological roles of circRNAs are currently a major focus of research attention. Multiple circRNAs, such as hsa_circ_0026123 [8], hsa_circ_0001068 [9], hsa_circ_0007874 [10] and hsa_circ_0025033 [11], participate in OC pathogenesis and may therefore serve as effective targets for diagnosis and treatment. In the current study, circRNA transcripts from OC and adjacent non-cancerous tissues were identified with the aid of Ribo-free RNA-Seq. Our results disclosed a significant increase in circ-PHC3 levels in OC tissues compared to control cell lines, supporting the utility of this circRNA as a prognostic biomarker for OC. Downregulation of circ-PHC3 led to suppression of OC malignant progression in both in vivo and in vitro, suggesting a critical function in cancer progression.

CircRNAs function as miRNA sponges to influence gene expression [12, 13]. Luciferase reporter experiments in our study demonstrated an association of circ-PHC3 with miR-497-5p. Downregulation of circ-PHC3 enhanced miR-497-5p expression while miR-497-5p inhibition led to recovery of the proliferation and invasion abilities of tumor cells under conditions of circ-PHC3 silencing. Recent studies have reported decreased expression of miR-497-5p OC [14]. Moreover, upregulation of miR-497-5p has been shown to suppress cell metastasis and proliferation in several cancer types, such as gastric cancer [15], squamous cell carcinoma [16], NSCLC [17], and hepatocellular cancer [18]. These findings suggest that miR-497-5p has anticancer effects.

Earlier studies have suggested that miR-497-5p interacts with SOX9 3’-UTR and inhibits its mRNA levels. Luciferase reporter analyses in the current investigation further validated SOX9 as a miR-497-5p target. Notably, miR-497-5p downregulation led to recovery of SOX9 expression after circ-PHC3 silencing. Moreover, overexpression of SOX9 rescued OC cell proliferation and invasion patterns under conditions of circ-PHC3 silencing. Sex-determining region Y (SRY)-box 9 protein (SOX9) is part of the SOX family of transcription factors (TF) that act as developmental regulators containing high mobility group (HMG) box DNA binding and transactivation domains [19]. SOX9 participates in multiple physiological activities, such as terminal differentiation and lineage restriction, via alterations in temporal and spatial expression patterns that vary among cell types and tissues [20]. Interestingly, SOX9 has been characterized as an oncogene in several tumor types, but also shown to function as a tumor suppressor [21, 22]. Previous findings suggest that SOX9 expressed by ectoderm- and endoderm-derived tissues in stem cell pools potentially regulates cancer stem cells (CSC) [23]. CSCs are associated with multiple functions, including cell proliferation, differentiation, migration, and angiogenesis [24], and their inhibition could therefore induce suppression of proliferation and invasion. Previous findings also find that the expression of SOX9 in OC tissues was upregulated which play an important role in regulates the chemoresistance of ovarian cancer cell to cisplatin-based chemotherapy [25]. In our experiments, circ-PHC3 silencing resulted in a decrease in the SOX9 stemness marker levels in OC cells. Notably, overexpression of SOX9 and miR-497-5p inhibition led to recovery of stemness in OC cells, suggesting that circ-PHC3 affects cancer stem cell differentiation through regulation of the miR-497-5p/SOX9 axis.

## Conclusion

In summary, circ-PHC3 influences OC cell invasion and proliferation patterns through exerting regulatory effects on miR-497-5p/SOX9 signaling. Further studies are needed to clarify how circ-PHC3 increases the stability of SOX9 at the post-translational modification level, which may be related to acetylation, phosphorylation or ubiquitination, etc. Taken together, our results strongly support the potential utility of circ-PHC3 as a promising candidate biomarker and therapeutic target for OC.

## Supplementary Information

**Additional file 1.**

## Data Availability

The datasets used and/or analyzed during the current study are available from the corresponding author on reasonable request.
